# Organoids in Pediatric Congenital Hepatobiliary Diseases: Current Status and Progress in Clinical Translational Research

**DOI:** 10.3390/biomedicines14061233

**Published:** 2026-05-29

**Authors:** Shanshan Zhang, Jingying Jiang, Shan Zheng

**Affiliations:** Department of Pediatric Surgery, Shanghai Key Laboratory of Birth Defect, and Key Laboratory of Neonatal Disease, Ministry of Health, Children’s Hospital of Fudan University, Shanghai 201102, China; m13577256586@163.com (S.Z.); jocelyn_17@163.com (J.J.)

**Keywords:** organoids, congenital hepatobiliary diseases, mechanisms and translation

## Abstract

Organoids are three-dimensional culture systems that self-organize and partially recapitulate the architecture, cellular composition, and functional properties of native tissues. In pediatric congenital hepatobiliary diseases, persistent cholestasis, bile duct maldevelopment, epithelial injury, and progressive fibrosis often lead to cirrhosis, liver failure, or the necessity for liver transplantation. Compared with conventional two-dimensional cell culture and animal models, hepatobiliary organoids provide patient-derived, human-relevant platforms for modeling disease mechanisms, evaluating therapeutic responses, and exploring regenerative strategies. Unlike previous reviews that mainly discuss general organoid culture systems or broad liver disease modeling, this review is organized around clinically oriented translational endpoints, including mechanistic target discovery, prognostic stratification, therapeutic validation, and regenerative reconstruction. We further discuss current barriers to clinical translation, including reproducibility, scalability, vascularization, immune integration, manufacturing standardization, and patient-specific genetic, environmental, and dietary modifiers. By integrating disease-specific mechanisms with translational applications, this review provides a framework for understanding how organoid-based platforms may contribute to future diagnosis, risk assessment, therapeutic decision-making, and regenerative medicine in pediatric congenital hepatobiliary disorders.

## 1. Introduction

**Figure 1:**
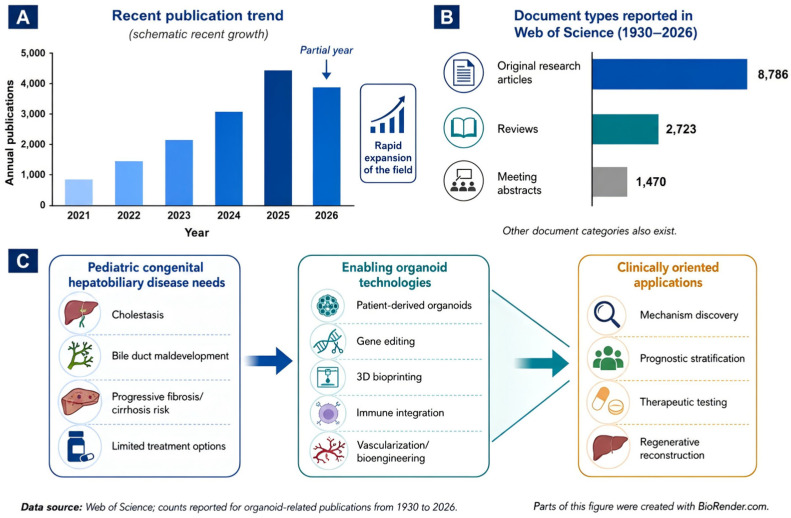
(**A**) Organoid-related research has increased steadily from 2021 to 2025, indicating rapid expansion of the field. (**B**) Web of Science records from 1930 to 2026 show that organoid-related publications are dominated by original research articles “8786” followed by reviews “2723” and meeting abstracts“1470”, reflecting both active experimental progress and growing academic discussion in the field. (**C**): Organoid technologies bridge unmet needs in pediatric congenital hepatobiliary diseases with clinically oriented applications, supporting mechanism discovery, prognostic stratification, therapeutic testing, and regenerative reconstruction. Parts of this figure were created with BioRender.com. Created in BioRender. Dong, R. (2026) https://BioRender.com/gr4ipv3 (accessed on 20 May 2026).

Organoids are stem cell-derived three-dimensional culture systems that self-organize and reproduce key structural and functional features of native tissues [1]. Since organoids were recognized by science as one of the major scientific breakthroughs of 2013 [2], the field has expanded rapidly. Bibliometric data from Web of Science show a marked increase in organoid-related publications over the past decade, particularly after 2020, reflecting the transition of organoid research from model establishment toward disease modeling, drug screening, regenerative medicine, and translational applications (Figure 1A). In addition to the growth in original research articles, the increasing number of review articles and conference abstracts further indicates active methodological development and academic communication in this field (Figure 1B).

Technological advances have further expanded the translational potential of organoids (Figure 1C). Three-dimensional bioprinting [3,4,5,6,7] enables spatial organization of cells and extracellular matrix components, thereby improving architectural control and supporting the construction of tubular or vascularized tissue-like structures. Gene-editing technologies allow pathogenic variants to be corrected or introduced in patient-derived organoids, providing platforms for functional validation and therapeutic testing [8,9,10,11]. Meanwhile, immune integration [12,13], stromal co-culture, vascularization strategies, and organ-on-a-chip systems are helping organoids more closely approximate the multicellular and dynamic microenvironment of human tissues. These advances are moving organoids beyond static in vitro models toward platforms for mechanism-based stratification, individualized drug testing [14,15], and regenerative reconstruction.

Congenital hepatobiliary diseases are primarily characterized by developmental abnormalities of the bile ducts and cholestasis as the central pathological features. Persistent or recurrent hepatobiliary injury may ultimately lead to progressive fibrosis and cirrhosis. Current therapeutic strategies remain largely dependent on surgical correction and liver transplantation [16,17,18]. By recapitulating patient-derived hepatobiliary tissue architecture and cell–cell interactions, organoids can model disease initiation and progression, as well as chronic cholestasis-induced injury, in a manner that more closely reflects in vivo pathophysiology [19]. Accordingly, recent advances in liver and biliary organoid systems have further expanded their applications from disease modeling and drug screening to regenerative medicine, gene therapy, and engineered multicellular platforms [20,21,22,23]. Organoid technology has therefore advanced research on congenital hepatobiliary diseases into a new stage, offering promising opportunities for individualized diagnosis and treatment as well as translational applications in regenerative medicine [24,25].

The novelty of this review lies in its disease-specific and translation-oriented framework. Rather than providing a general overview of hepatobiliary organoid culture systems, we synthesize recent evidence across four representative pediatric congenital hepatobiliary diseases: biliary atresia, Alagille syndrome, polycystic liver disease, and Wilson’s disease. We compare how organoids contribute to mechanistic target discovery, prognostic prediction, therapeutic validation, and regenerative reconstruction in each disease context. This structure allows the current maturity, translational readiness, and unresolved bottlenecks of different organoid models to be evaluated side by side.

## 2. History and Development of Organoid Technology

The conceptual origins of organoid research can be traced to 1907, when Wilson et al. observed that dissociated sponge cells could spontaneously reaggregate and reorganize in vitro, providing early evidence of cellular self-organization [26]. In the 1980s, the development and characterization of extracellular matrix components, including collagen and laminin, enabled the construction of three-dimensional tissue architectures and highlighted the instructive role of the matrix microenvironment in regulating cell behavior [27,28,29]. With the maturation of pluripotent stem cell technologies in the 21st century, organoid-based models increasingly became important tools for studying human development and disease [30,31]. A major milestone occurred in 2009, when Sato et al. established long-term expandable intestinal organoids, demonstrating that adult stem cells could generate stable, self-renewing, and differentiated epithelial structures in vitro [32].

Since then, organoid systems have been developed for multiple tissues, including the liver, biliary tract, esophagus, and heart [33]. More recently, the integration of CRISPR-based gene editing, single-cell transcriptomics, spatially resolved analytical methods, immune co-culture, and bioengineering strategies has substantially improved the precision, reproducibility, and functional complexity of organoid models [34,35]. By 2025, fully human liver immune organoid platforms had been reported to predict immune-mediated drug-induced liver injury, underscoring the potential of organoids for precision drug safety assessment and clinical translation [36].

In recent years, Organoids have evolved from experimental disease models to platforms for therapeutic validation, tissue repair, and regenerative reconstruction. Patient- or healthy-donor-derived organoids can be expanded in vitro and transplanted into injured tissues, where they may differentiate in situ and support structural restoration and functional recovery [8,34,37]. In parallel, the convergence of organoid biology and three-dimensional bioprinting has enabled more precise spatial organization of cells, extracellular matrix components, and bioinks [38]. Compared with conventional organoids that rely primarily on spontaneous self-organization, bioprinting can generate tubular constructs, complex luminal networks, and larger-scale tissue equivalents [39,40]. Strategies incorporating endothelial or mesenchymal cells may further promote microvascular network formation, enhance organoid maturation, and improve long-term graft stability [41,42].

Organoids also provide patient-derived platforms for validating gene therapy and gene-correction strategies, particularly in inherited hepatobiliary disorders. Since patient-derived organoids preserve individual mutational backgrounds, they can be used to evaluate genome-editing tools, such as base editing and prime editing, and to assess whether genetic correction restores disease-relevant structure and function [43,44]. CRISPR-mediated knockout and knock-in approaches further facilitate pathogenic variant interpretation, disease-pathway dissection, and preclinical evaluation of corrected organoids for potential transplantation [45]. Collectively, organoid technology has progressed from a model of cellular self-organization to an enabling platform for disease modeling, precision therapeutic testing, gene correction, and regenerative medicine. These advances are summarized in Table 1.

## 3. Current Status of Organoid Research in the Hepatobiliary System

Hepatic and biliary organoids have emerged as important experimental platforms for investigating hepatobiliary diseases, regenerative medicine, and genome-editing strategies [47]. Liver organoids recapitulate key hepatic parenchymal functions, including metabolism, protein secretion, and detoxification [37,48], whereas cholangiocyte organoids are mainly used to model epithelial polarity, lumen formation, and biliary regenerative responses [49,50]. Together, these complementary systems provide a foundation for studying hepatobiliary lineage specification, disease mechanisms, and functional reconstruction.

Liver organoid research has progressed from methodological establishment toward translational development. In 2013, induced pluripotent stem cell (iPSC)-derived liver bud models were established [51], providing an experimental basis for studying hepatobiliary specification, inherited hepatobiliary disorders, and multi-lineage differentiation systems. Between 2021 and 2026, advances in genetic engineering, vascularization, and tissue engineering enabled the generation of more mature liver organoids with sinusoid-like networks. These models better recapitulate inherited liver diseases, metabolic dysfunction-associated steatotic liver disease, and liver fibrosis, while supporting disease-relevant drug screening and selected transplantation-based functional improvement [52,53,54,55,56,57,58,59,60,61,62].

In parallel, bile duct scaffold research has evolved from natural matrices and conventional synthetic polymers toward advanced biomaterials and three-dimensional (3D) printing technologies [63,64]. Early studies used decellularized porcine vascular matrices or polycaprolactone (PCL)/polydioxanone (PDO) composite scaffolds [65]. When combined with biliary epithelial cells or mesenchymal stem cells (MSCs), these constructs supported segmental bile duct regeneration and biliary injury repair in large-animal models [66]. More recently, 3D printing has enabled multifunctional and patient-specific biliary scaffolds, including biodegradable polyurethane-based composites [67,68,69], thereby improving scaffold biocompatibility, anatomical precision, and the feasibility of individualized biliary reconstruction. The detailed discovery framework is shown in Table 2.

Overall, hepatobiliary organoids are moving from simple epithelial models toward engineered, multicellular, and scaffold-integrated systems. However, their clinical translation still requires further advances in organoid-scaffold integration, vascularization, immune incorporation, functional maturation, long-term safety evaluation, reproducibility, and standardized manufacturing scalability.

## 4. Translational Progress of Organoid Research in Pediatric Congenital Hepatobiliary Disease

**Figure 2:**
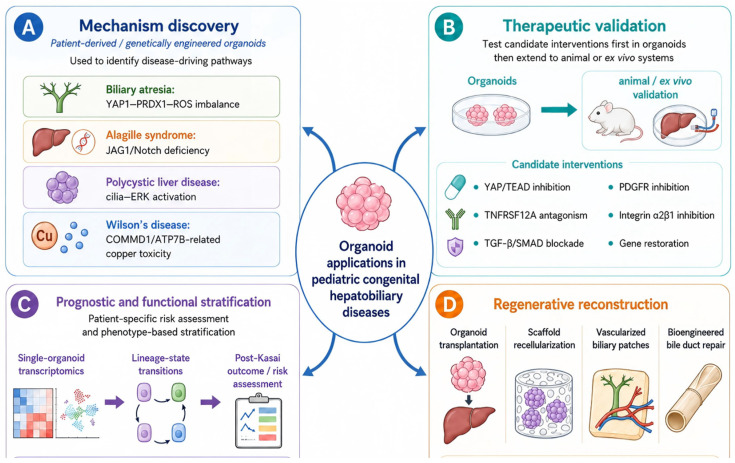
Mechanistic and translational applications of organoids in pediatric congenital hepatobiliary diseases: (**A**) Patient-derived or genetically engineered organoids to identify disease-driving pathways in BA, ALGS, PLD, and Wilson’s disease. (**B**) Therapeutic validation in which candidate interventions are first tested in organoids and then extended to animal or ex vivo models. (**C**) Prognostic and functional stratification based on single-organoid transcriptomics, lineage-state transitions, and post-Kasai risk assessment. (**D**) Regenerative reconstruction strategies, including organoid transplantation, scaffold recellularization, vascularized biliary patches, and bioengineered bile duct repair. Parts of this figure were created with BioRender.com. Created in BioRender. Dong, R. (2026) https://BioRender.com/as6oyoi (accessed on 20 May 2026).

Organoid applications in pediatric congenital hepatobiliary diseases can be understood through four major translational routes: mechanism discovery, prognostic stratification, therapeutic validation, and regenerative reconstruction. The maturity of these routes differs substantially among diseases. Biliary atresia has generated the most direct evidence linking patient-derived organoids to target identification and outcome prediction, whereas Alagille syndrome models mainly highlight developmental signaling and region-specific biliary regeneration. Polycystic liver disease organoids are particularly useful for modeling cystogenesis and supporting antifibrotic drug screening, while Wilson’s disease models provide a clearer framework for gene correction, copper-toxicity testing, and autologous cell-based therapy. The specific mechanisms are illustrated in Figure 2.

### 4.1. Biliary Atresia

Biliary atresia (BA) is the most common individual cause of neonatal cholestasis and remains the leading indication for pediatric liver transplantation worldwide. Accumulating evidence indicates that organoid applications in BA have expanded from pathological modeling and mechanistic investigation to prognostic stratification and exploratory regenerative or cell-based therapeutic strategies.

#### 4.1.1. Organoid-Informed Therapeutic Targeting and Antifibrotic Strategies for Biliary Atresia

Xie et al. (2024) found that epithelial cell adhesion molecule-positive (EpCAM^+^) cholangiocytes in human and rhesus rotavirus (RRV)-induced mouse BA tissues exhibited increased Yes-associated protein 1 (YAP1) expression and nuclear localization, suggesting aberrant Hippo-YAP1 activation [71]. By establishing intrahepatic cholangiocyte-like cell (CLC) organoids derived from mouse hepatic EpCAM^+^ cells, the study further demonstrated that YAP1 activation led to smaller organoids with impaired expansion and abnormal multilocular morphology. Conversely, treatment with the YAP/transcriptional enhanced associate domain (TEAD) inhibitory peptide, Peptide17, partially restored the cystic morphology of organoids derived from RRV-induced BA mice, reduced reactive oxygen species (ROS) levels by approximately 40%, decreased cholangiocyte apoptosis, and restored keratin 19 (KRT19) expression. Thus, the organoid experiments converted the observation of YAP1 dysregulation in BA tissues into functional evidence of impaired cholangiocyte-like cell development, suggesting that the YAP1-ROS axis may contribute to aberrant biliary repair and regeneration in BA.

Xiao et al. (2025) integrated single-cell and spatial transcriptomic analyses to identify a pathogenic cholangiocyte-enriched niche and nominated TNFSF12-TNFRSF12A as a key ligand–receptor interaction in BA [72]. However, these omics-based analyses mainly provided correlative evidence. The subsequent organoid experiments functionally validated this predicted signaling axis in a human cholangiocyte-based system. TNFSF12 stimulation did not markedly alter organoid morphology but induced a disease-specific inflammatory response in BA-derived biliary organoids, characterized by increased C-C motif chemokine ligand 2 (CCL2) production and secretion. Culture supernatants from TNFSF12-treated BA organoids promoted monocyte migration, which was blocked by a C-C motif chemokine receptor 2 (CCR2) antagonist. Thus, the organoid model bridged spatial transcriptomic discovery and animal therapeutic validation by demonstrating that BA cholangiocytes can actively recruit monocytes through the TNFSF12-TNFRSF12A-CCL2-CCR2 axis.

In the same year, Ayabe et al. and Chusilp et al. used cholangiocyte organoid-based systems to implicate transforming growth factor-β (TGF-β)-dependent epithelial-mesenchymal transition (EMT) as a driver of BA-associated hepatic fibrosis [38,63]. Ayabe et al. combined human-derived cholangiocyte organoids with an RRV-induced BA rodent model and showed that inhibition of TGF-β/Activin-SMAD2/3 signaling suppressed EMT, promoted cholangiocyte maturation, and mitigated bile duct injury and fibrosis. Chusilp et al. used a TGF-β1-induced fibrotic liver organoid model and reported that co-culture with human amniotic fluid stem cells (hAFSCs) partially restored CK19 and E-cadherin expression, reduced mesenchymal marker expression, and decreased collagen type I alpha 1 chain (COL1A1) levels, consistent with an antifibrotic effect. Together, these studies support targeting the TGF-β-EMT axis as a potential antifibrotic strategy in BA; however, the hAFSC-based approach remains at the in vitro proof-of-concept stage.

#### 4.1.2. Organoid Lineage Conversion Predicts Post-Kasai Outcomes and Cholangitis Risk

In a retrospective cohort study, Wai et al. (2024) applied patient-derived liver organoids to prognostic stratification after Kasai portoenterostomy [73]. The study included 32 liver tissue specimens and generated a single-organoid transcriptomic dataset comprising 70 portoenterostomy (at-KPE) organoids, 112 after surgery (post-KPE) organoids, and 47 control organoids. At-KPE BA organoids showed increased hepatocyte-associated transcriptional features, whereas post-KPE organoids from native liver survivors exhibited reduced hepatocyte-associated features and increased cholangiocyte-associated features, indicating a hepatocyte-to-cholangiocyte transcriptional transition associated with biliary recovery. In contrast, organoids from patients who ultimately underwent liver transplantation retained relatively high hepatocyte-associated features, suggesting persistent lineage dysregulation and disease progression. These findings indicate that organoid-based transcriptomic profiling may provide a patient-specific, lineage-based readout of biliary repair capacity and post-Kasai outcome, although validation in larger adverse-outcome cohorts remains necessary. However, the small number of adverse-outcome samples in the LTR group (*n* = 3) limits the generalizability of these findings. However, these findings are consistent with those of Amarachintha et al. (2022), who reported that biliary atresia cholangiocyte-like organoids (BACOs) exhibit cholangiocyte developmental arrest, resulting in impaired epithelial barrier function [74]. Together, these organoid-based studies suggest that the combined assessment of dysregulated lineage transition and epithelial barrier dysfunction may provide a useful prognostic framework for stratifying post-Kasai disease progression and cholangitis risk.

#### 4.1.3. Cholangiocyte Organoid Transplantation and Scaffold-Engineered Reconstruction

Sampaziotis et al. (2021) advanced cholangiocyte organoid research from in vitro disease modeling to regenerative repair [52]. By demonstrating that cholangiocyte organoids can be expanded in vitro and regain region-specific biliary features after transplantation, the study addressed the limited availability and poor expandability of primary cholangiocytes. In a mouse model of biliary injury, transplantation of gallbladder-derived cholangiocyte organoids repaired intrahepatic bile duct injury and extended survival from less than 3 weeks in controls to approximately 3 months, indicating functional rather than merely phenotypic repair. Importantly, in an ex vivo human liver normothermic machine perfusion (NMP) model, extrahepatic bile duct organoids engrafted into ischemia-injured bile ducts, restored epithelial continuity, and were associated with improved bile pH and bile output. These experiments bridged the translational gap between animal models and human biliary repair. Related scaffold-based studies further showed that bile-derived or extrahepatic cholangiocyte organoids can repopulate decellularized bile duct scaffolds and form bile duct-like structures with mature cholangiocyte markers, epithelial barrier function, transepithelial electrical resistance (TEER), and ion channel activity [75,76]. Together, these findings support an engineering-based strategy in which expandable cholangiocyte organoids are combined with biliary scaffolds to reconstruct damaged extrahepatic bile ducts, offering a potential alternative cell source and regenerative pathway for extrahepatic biliary diseases, including biliary atresia (BA). Table 3 summarizes the translational applications of organoid-based research in biliary atresia.

### 4.2. Alagille Syndrome

Alagille syndrome (ALGS) is an autosomal dominant congenital multisystem disorder characterized by intrahepatic bile duct paucity and progressive cholestasis.

#### 4.2.1. Therapy Outcomes: JAG1/Notch–Targeted Exogenous Modulation

In ALGS-related studies, organoid experiments helped bridge developmental findings from animal models with human or region-specific biliary responses. Zhao et al. (2022) showed in a jag1b/2b-deficient zebrafish ALGS-like model that restoration of Jagged/Notch signaling promoted intrahepatic biliary repair [77]. Human cholangiocyte-related organoids further demonstrated that the Notch agonist NoRA1 could induce SOX9 expression, supporting the translational relevance of pharmacological Notch–SOX9 activation in human biliary-lineage cells. However, these organoids were not patient-specific ALGS organoids and, therefore, mainly provided human-system validation rather than direct disease modeling. In contrast, Iqbal et al. (2024) established region-specific hilar intrahepatic bile duct organoids (HICOs) and peripheral intrahepatic bile duct organoids (PICOs) from a Jag1 mutant mouse model [78]. These organoids revealed that JAG1 deficiency reduced proliferation in both compartments, but most prominently in HICOs, and induced attenuated Notch activity with a hepatocyte-like transcriptional shift marked by HNF4A positivity. Importantly, insulin-like growth factor 1 (IGF1) rescued proliferation and survival only in PICOs. Thus, organoid experiments overcame the limitations of whole-animal phenotyping by separating compartment-specific biliary defects and therapeutic responsiveness, indicating that ALGS regenerative strategies may need to be tailored to distinct biliary regions.

#### 4.2.2. Tissue Regeneration: Restoring Biliary Continuity and Function via Vascularized Patches

Carolina et al. (2024) advanced biliary organoid engineering by incorporating human induced pluripotent stem cell (hiPSC)-derived vascular components into liver organoids to generate blood vessel-incorporated liver organoids (BVLOs) [79]. This strategy addressed a major limitation of conventional epithelial-only biliary organoids, which poorly recapitulate the spatial interaction between developing intrahepatic bile ducts and portal vein-like vascular structures. In BVLOs, hiPSC-derived liver progenitors differentiated into cholangiocytes and acquired epithelial features, including intercellular junctions, apical microvilli, and secretory activity. Functionally, the biliary epithelium exhibited multidrug resistance protein 1 (MDR1)-mediated fluorescent substrate efflux and cystic fibrosis transmembrane conductance regulator (CFTR)-dependent luminal swelling, supporting the formation of functionally competent bile duct-like structures. Single-cell RNA sequencing further suggested that bile duct–blood vessel interactions were mediated partly through transforming growth factor β (TGFβ) and Notch signaling, and JAG1 knockout in the vascular compartment impaired bile duct formation. After transplantation onto the liver surface of cholestatic mice, BVLOs established luminal continuity with host intrahepatic bile ducts and were associated with short-term alleviation of cholestasis. Together, these findings provide a methodological foundation for mechanistic studies of Alagille syndrome (ALGS) and the development of engineered strategies for biliary reconstruction.

### 4.3. Polycystic Liver Disease

Polycystic liver disease (PLD) is a congenital ductal plate malformation associated with pathogenic genetic variants, in which dysregulated luminal fluid secretion and cholangiocyte proliferation promote cyst expansion and progressive liver injury (Table 4).

#### 4.3.1. Therapeutic: Fibrosis-Targeted Therapy and Pathway-Axis Intervention

In PLD-related studies, organoid experiments provided controlled platforms to link cystic cholangiopathy, fibrotic remodeling, and candidate therapeutic pathways. Guan et al. (2021) generated human induced pluripotent stem cell (iPSC)-derived multilineage liver organoids that recapitulated key ARPKD-associated liver phenotypes, including abnormal bile duct architecture, collagen deposition, and fibrosis-like remodeling within 21 days [54]. By combining single-cell RNA sequencing (scRNA-seq) and cytometry by time-of-flight (CyTOF), the authors identified platelet-derived growth factor receptor β-signal transducer and activator of transcription 3-positive (PDGFRβ-STAT3^+^) myofibroblasts as a central profibrotic population, and pharmacological inhibition of platelet-derived growth factor receptor (PDGFR) attenuated the fibrotic phenotype. Thus, this multilineage organoid model overcame the limitations of epithelial-only systems by integrating cholangiocyte abnormalities with mesenchymal activation and enabling target validation in a human multicellular context.

In parallel, Chen et al. (2022) combined a conditional Kif3a knockout mouse model with thioacetamide (TAA)-induced injury and cholangiocyte progenitor organoids to dissect the role of primary cilia in cystic remodeling [80]. Loss of Kif3a in CK19^+^ biliary epithelial cells did not substantially exacerbate global hepatic fibrosis but selectively promoted microcystic lesions and proliferation of cholangiocyte progenitor organoids, accompanied by sustained extracellular signal-regulated kinase (ERK) activation. These findings refined the interpretation of ciliary dysfunction by suggesting that primary cilia loss preferentially drives ductular cystic proliferation rather than generalized fibrogenesis. Together with the Ca^2+^-adenylyl cyclase 5 (AC5)-cyclic adenosine monophosphate (cAMP)-ERK pathway previously implicated in PLD cyst growth, these organoid-based studies provide mechanistic bridges between clinical cystic phenotypes and targetable signaling nodes, including cilia/ERK and PDGFRβ/STAT3.

#### 4.3.2. Preliminary Screening of Candidate Therapeutics

Waddell et al. (2023) used liver organoids to functionally dissect a TGFβ-extracellular matrix (ECM)–integrin signaling axis in polycystic liver disease (PLD) [83]. By culturing primary bile duct tissue and biliary epithelial cells (BECs) from genetically engineered mice and patients with PLD in Matrigel, the authors converted the structural remodeling of the bile duct from a static histological observation into a dynamic and quantifiable in vitro process. Wild-type bile duct tissue spontaneously transitioned from tubular structures to spherical cysts within 72 h, allowing cyst area, epithelial proliferation, and tubular-to-cystic conversion to be directly measured. Pharmacological inhibition of SMAD3 with SIS3 suppressed cystogenesis, reduced cyst area, and decreased fibronectin 1 (FN1) production, supporting a causal role for TGFβ/SMAD3-driven ECM synthesis in cyst formation. In parallel, genetic loss of integrin subunit α2 (ITGA2) or pharmacological inhibition of integrin α2β1 with TC-I15 impaired cyst growth and mature cyst formation. Notably, organoids derived from patients with PLD appeared more sensitive to TC-I15, suggesting disease-selective therapeutic potential. Thus, the organoid experiments overcame the limitations of static tissue analysis and whole-animal models by providing a controllable human-relevant platform to validate the TGFβ/SMAD3-FN1-integrin α2β1 axis as a functional driver of biliary cystogenesis and a candidate therapeutic target.

### 4.4. Wilson’s Disease (WD)

Wilson’s disease (WD) is an inherited disorder of copper metabolism caused by pathogenic variants in copper-handling genes and characterized by intrahepatocellular copper accumulation.

#### 4.4.1. Therapeutic: Genetic Rescue to Decoppering Drug Screening

Nantasanti et al. (2015) established long-term expandable canine liver organoids from liver biopsies of dogs with naturally occurring COMMD1 deficiency, a large-animal model of hepatic copper accumulation [84]. These organoids recapitulated copper accumulation and copper-induced toxicity in vitro, while lentiviral restoration of COMMD1 reversed cellular susceptibility to high-copper exposure. This work provided large-animal proof-of-concept evidence spanning disease modeling and gene restoration and outlined a technical prototype for the autologous reinfusion of genetically corrected organoids. For drug screening and efficacy assessment, Kim et al. (2020) introduced a common ATP7B mutation (R778L) into normal human embryonic stem cells using CRISPR/Cas9 and then differentiated these cells into hepatocyte-like cells [85]. The mutant cells showed greater loss of viability under copper loading and exhibited transcriptomic reprogramming, enabling systematic evaluation of the protective effects of chelators such as D-penicillamine and trientine. This strategy established a genotype-controlled human pluripotent stem cell-derived WD model suitable for therapeutic screening and response profiling.

#### 4.4.2. Regenerative: Functional Repair and Long-Term Persistence

Kruitwagen et al. (2024) extended COMMD1-deficient liver organoid research from in vitro disease modeling to large-animal transplantation [82]. By generating autologous liver organoids from COMMD1-deficient dogs, restoring COMMD1 expression by lentiviral transduction, and expanding the corrected cells to transplantable numbers within 12 weeks with high viability, the study addressed the key translational limitation of whether gene-corrected organoid-derived cells can be manufactured at a clinically relevant scale. Repeated portal vein infusion and intrahepatic injection further demonstrated procedural feasibility in a large-animal model that closely resembles human Wilson disease. Corrected cells persisted in the liver for up to 2 years without tumor formation, supporting long-term survival and apparent safety. However, engraftment and liver repopulation remained low, and functional recovery of copper excretion was not achieved. Thus, this study provided important preclinical evidence for the feasibility and safety of autologous, gene-corrected liver organoid-derived cell transplantation, while also highlighting engraftment efficiency and functional restoration as major barriers to clinical translation in Wilson disease. These two aspects are summarized in Table 5.

## 5. Limitations and Future Perspectives

Despite rapid progress, several limitations of hepatobiliary organoid technologies remain insufficiently resolved and continue to restrict clinical translation. Reproducibility is limited by differences in cell sources, extracellular matrices, growth factor cocktails, culture duration, and maturation criteria, leading to inter-batch and interlaboratory variability [51,83,86,87]. Scalability is also constrained by continued reliance on poorly defined matrices such as Matrigel and by limited control over matrix stiffness, organoid geometry, nutrient transport, and automated production. In addition, most current systems incompletely reproduce the pediatric hepatobiliary microenvironment, particularly vascular perfusion, stromal organization, immune-cell interactions, and bile flow-related dynamics, which are essential for modeling inflammation, fibrosis, immune-mediated cholangiopathies, and functional maturation [83,86]. Although vascularized organoids, organoid-on-chip platforms, and multicellular co-culture systems are emerging, long-term vascular stability, immune integration, physiological bile drainage, and functional maturation remain challenging. Moreover, the clinical predictive value of organoid-based drug screening, prognostic modeling, therapeutic validation, and regenerative repair remains largely preclinical and requires validation in larger longitudinal cohorts that incorporate patient-specific genetic, environmental, microbiota-related, and dietary modifiers. Finally, regenerative applications still face unresolved issues, including engraftment efficiency, long-term safety, immune consequences, delivery routes, quality control, and regulatory requirements [70]. Future studies should therefore prioritize standardized, scalable, vascularized, immune-competent, and clinically validated organoid platforms to enable reliable translation into precision medicine and regenerative therapy for pediatric congenital hepatobiliary diseases.

Although many infantile hepatobiliary disorders are initiated by monogenic defects, genotype alone does not fully determine disease severity or clinical trajectory. Phenotypic expression may be modified by genetic background, secondary modifier genes, developmental stage, inflammatory status, microbiota-related signals, environmental exposures, and diet. For example, Alagille syndrome shows marked clinical variability even among individuals within the same family, indicating that JAG1/NOTCH2 mutations are shaped by additional modifiers. Similarly, Wilson’s disease is influenced not only by ATP7B mutations but also by genetic and environmental modifiers, including copper intake and other copper-handling pathways [88,89]. Therefore, future organoid studies should move beyond single-gene modeling and incorporate multi-layered experimental designs. Patient-derived organoids should be established from genetically and clinically stratified cohorts, ideally including patients with the same causal mutation but divergent phenotypes. Recent studies have established patient-derived liver organoid platforms from healthy, steatotic, and cirrhotic human liver tissues, supporting the feasibility of tissue-derived organoids as scalable translational models for liver disease research [90]. Isogenic gene-corrected controls, CRISPR-edited mutation-introduction models, and multi-omics profiling can help distinguish primary mutation-driven effects from background-dependent modifiers. In parallel, organoids should be exposed to disease-relevant extrinsic factors, such as bile acids, copper, inflammatory cytokines, microbial metabolites, hypoxia, or diet-related nutrients, to model gene–environment interactions. More physiologically complete systems, including co-culture with immune, mesenchymal, endothelial, and microbiome-related components, may further clarify how external and microenvironmental signals reshape monogenic disease phenotypes. In this way, organoids can serve not only as models of inherited mutations but also as controllable platforms for dissecting why the same genotype produces variable disease severity and treatment responses [91].

## 6. Conclusions

Organoid technologies are reshaping the study of congenital hepatobiliary diseases by providing patient-specific, human-relevant models that capture key aspects of developmental defects, genetic injury, cholestasis, inflammation, and fibrosis. In biliary atresia, organoids help clarify cholangiocyte dysfunction, inflammatory amplification, lineage dysregulation, and post-Kasai outcome heterogeneity. In inherited cholangiopathies and metabolic liver diseases, they enable mutation-specific modeling, therapeutic screening, and gene-correction assessment. Together with advances in vascularization, immune integration, bioengineering, and genome editing, hepatobiliary organoids are evolving from experimental culture systems into translational platforms for mechanism discovery, precision therapy, prognostic stratification, and regenerative repair in pediatric congenital hepatobiliary diseases.

## Figures and Tables

**Table 1:** Historical milestones in organoid research and technology.

Stage	Research Priorities	Typical Outputs	Ref.
Early development (1907–1986)	Cellular self-organization and reaggregation	Single cells reconstitute tissue-like structures.	[26,46]
	Extracellular Matrix (ECM)-cell adhesion as an instructive microenvironment	Mechanistic link between ECM adhesion and cellular phenotype regulation	[28]
Mid-stage consolidation (2009–2020)	Reproducible organoid culture systems	Establishment of expandable, differentiable, self-organizing organoid cultures	[32]
	Systematization of ECM and the 3D paradigm	Frameworks formalizing ECM roles and the 3D experimental paradigm	[27,29]
	Organoid modeling framework and disease-modeling boundaries	Definition of applications, limitations, and standardization challenges for organoids	[31,34]
Current hotspots (2021–2025)	Engineered organoids, dynamic systems, multicellular niches, genome editing	Expansion of iPSC-based, organ-specific human models for complex phenotypes	[30,33]
	Dynamic system integration (organ-on-a-chip)	Perfused chip platforms enhance physiologically relevant functional readouts	[25]
	Multicellular niches and immune integration	Immune-organoid co-culture enables functional immune-response screening	[35]
	Biomanufacturing and materials (bioprinting/vascularization)	Bioprinting builds perfusable constructs and supports vascularization studies	[39,40,41,42]
	Precision therapeutic validation (genome editing)	Genome editing corrects mutations and restores function in patient organoids	[43,44]
	From mechanism to targets and hepatobiliary translation framework	Therapeutic target and translational frameworks	[36,37,38]

**Table 2:** Impact of organoids on translational research in hepatobiliary diseases.

Translational Direction	Representative Studies	Impact	Ref.
Diagnostics	Sampaziotis (2015) & Ogawa (2015): stable cholangiocyte organoids from iPSCs	Human-relevant bile duct model for mechanistic	[52,53,54,55]
	Guan (2017) & Andersson (2018): Notch disruption → bile duct defects; CRISPR rescues structure		
Prognostic Evaluation	Hendriks (2023): fetal liver organoids recapitulate steatosis for drug testing	Fibrosis/steatosis organoid models guide treatment and prognosis.	[60,62]
	Mochida (2025): organoids recapitulate injury-induced fibrosis via IL-1β-mediated cell crosstalk		
Regeneration	Velazquez (2021): vascularization engineering improves liver bud maturation and functional engraftment	Engineered vascularized and multicompartment organoids support functional repair, toxicity testing, and regenerative translation.	[59,61,65,69,70]
	Saiki (2024): sinusoid-like vascularized organoids rescue hemophilia A phenotype		
	Strucker (2016) & Cordista (2024): scaffolds promote vascular and biliary lining formation		
	Qin et al. (2026): cadherin-engineered poly(lactic-co-glycolic acid) microspheres guided mesenchymal stem/stromal cells into multilineage liver organoids.		
Therapy	Guan (2017): Gene editing rescues bile duct function	Targeted therapy gene therapy Cell-based functional replacement Tissue-engineered structural repair	[54,66,68]
	Zong (2017) & Xiang Y (2020): scaffolds promote bile duct epithelial layer formation and repair		

**Table 3:** Translational applications of organoid research in biliary atresia.

Direction	Key Findings	Technical Advances	Inherent Limitations	Ref.
Therapeutic targeting	YAP1–PRDX1–ROS TNFSF12–TNFRSF12A TGF-β/Activin-SMAD2/3-driven EMT	Organoid-based functional validation and animal testing	Limited validation of efficacy, safety, and microenvironmental complexity.	[38,63,71,72]
Prognostic stratification	Post-Kasai outcome stratification and risk assessment	Single-organoid transcriptomic profiling	Small cohorts; limited prospective and multicenter validation.	[73,74]
Regenerative reconstruction	Repair and bioengineered reconstruction	Cell sourcing, scaffold recellularization, and functional assessment	Mainly ex vivo or preclinical; standardization and clinical translation remain challenging.	[22,75,76]

**Table 4:** Translational applications of organoid research in polycystic liver disease.

Category	Model Type	Disease Application	Technical Advances	Inherent Limitations	Ref.
Therapeutic	Human iPSC-derived multi-lineage liver organoids	Fibrosis-targeted therapy and pathway-axis intervention	scRNA-seq and CyTOF	limited in vivo validation	[54,80,81]
	Kif3a knockout mouse model plus cholangiocyte progenitor organoids	Cilia–ERK signaling axis in cystogenesis	vivo–organoid combined mechanistic validation	More relevant to microcystic lesions than full PLD progression	
Screening of candidate therapeutics	Mouse and human bile duct–derived organoids	Preliminary screening of candidate therapeutics	Cross-species cystogenesis platform; morphologic and protein-array evaluation	Predominantly in vitro; lacking long-term and in vivo validation	[82]

**Table 5:** Translational applications of organoid research in Wilson’s disease.

Category	Model Type	Disease Application	Technical Advances	Inherent Limitations	Ref.
Therapeutic	Canine liver biopsy-derived organoids from naturally occurring COMMD1 deficiency	Genetic rescue and proof-of-concept disease modeling	Long-term expandable large-animal organoids; lentiviral COMMD1 restoration	Large-animal model; limited direct human validation	[84,85]
	CRISPR/Cas9-engineered hESC-derived hepatocyte-like cells carrying ATP7B R778L	Decoppering drug screening and efficacy assessment	Genotype- controlled hPSC-WD platform for chelator testing and response profiling	Hepatocyte-like cell model; limited regenerative and in vivo validation	
Regenerative	Autologous gene-corrected liver organoid cells in COMMD1-deficient dogs	Functional repair and long-term persistence	Portal vein infusion and intrahepatic delivery of corrected organoid cells; long-term in vivo persistence	Small sample size; canine model; human clinical applicability remains unproven	[82]

## Data Availability

No new data were created or analyzed in this study.

## Abbreviations

Abbreviation	Full term
iPSCs	induced pluripotent stem cells
ASCs	adult stem cells
PSC	pluripotent stem cell
CRISPR	clustered regularly interspaced short palindromic repeats
3D	three-dimensional
ECM	extracellular matrix
PCL	polycaprolactone
PDO	polydioxanone
MSCs	mesenchymal stem cells
BA	biliary atresia
EpCAM^+^	epithelial cell adhesion molecule–positive
PRDX1	peroxiredoxin 1
ROS	reactive oxygen species
KRT19	keratin 19
HiBECs	human intrahepatic biliary epithelial cell organoids
aHSCs	activated hepatic stellate cells
TGF-β	transforming growth factor-β
EMT	epithelial–mesenchymal transition
hAFSCs	human amniotic fluid stem cells
COL1A1	collagen type I alpha 1 chain
NLS	native liver survival
LTR	liver transplantation required
APRi	aspartate aminotransferase to platelet ratio index
GGT	gamma-glutamyltransferase
BACOs	biliary atresia cholangiocyte-like organoids
NMP	normothermic machine perfusion
TEER	transepithelial electrical resistance
ALGS	Alagille syndrome
HICOs	hilar intrahepatic bile duct organoids
PICOs	peripheral intrahepatic bile duct organoids
HNF4A+	hepatocyte nuclear factor 4α positivity
IGF1	insulin-like growth factor 1
BVLO	vascular-integrated biliary/liver organoid
ECs	endothelial cells
MDR1	multidrug resistance protein 1
CFTR	cystic fibrosis transmembrane conductance regulator
PLD	polycystic liver disease
ARPKD	autosomal recessive polycystic kidney
scRNA-seq	single-cell RNA sequencing
CyTOF	cytometry by time-of-flight
PDGFR	platelet-derived growth factor receptor
TAA	thioacetamide
ERK	extracellular signal-regulated kinase
BEC	bile duct epithelial cells
WD	Wilson’s disease
